# Exosomes from human urine‐derived stem cells enhanced neurogenesis via miR‐26a/HDAC6 axis after ischaemic stroke

**DOI:** 10.1111/jcmm.14774

**Published:** 2019-10-31

**Authors:** Xiaozheng Ling, Guowei Zhang, Yuguo Xia, Qingwei Zhu, Juntao Zhang, Qing Li, Xin Niu, Guowen Hu, Yunlong Yang, Yang Wang, Zhifeng Deng

**Affiliations:** ^1^ Department of Neurosurgery Shanghai Jiao Tong University Affiliated Sixth People' Hospital Shanghai China; ^2^ Department of Neurosurgery Tai'an City Central Hospital Tai'an China; ^3^ Department of Neurosurgery Shanghai First People's Hospital Shanghai Jiao Tong University School of Medicine Shanghai China; ^4^ Institute of Microsurgery on Extremities Shanghai Jiao Tong University Affiliated Sixth People' Hospital Shanghai China

**Keywords:** exosome, histone deacetylase 6, human urine‐derived stem cell, microRNA‐26a, neural stem cell

## Abstract

Endogenous neurogenesis holds promise for brain repair and long‐term functional recovery after ischaemic stroke. However, the effects of exosomes from human urine‐derived stem cells (USC‐Exos) in neurogenesis remain unclear. This study aimed to investigate whether USC‐Exos enhanced neurogenesis and promoted functional recovery in brain ischaemia. By using an experimental stroke rat model, we found that intravenous injection of USC‐Exos enhanced neurogenesis and alleviated neurological deficits in post‐ischaemic stroke rats. We used neural stem cells (NSCs) subjected to oxygen‐glucose deprivation/reoxygenation (OGD/R) as an in vitro model of ischaemic stroke. The in vitro results suggested that USC‐Exos promoted both proliferation and neuronal differentiation of NSCs after OGD/R. Notably, a further mechanism study revealed that the pro‐neurogenesis effects of USC‐Exos may be partially attributed to histone deacetylase 6 (HDAC6) inhibition via the transfer of exosomal microRNA‐26a (miR‐26a). Taken together, this study indicates that USC‐Exos can be used as a novel promising strategy for brain ischaemia, which highlights the application of USC‐Exos.

## 1 INTRODUCTION

Stroke is a major cause of mortality and long‐term disability worldwide.^1^, ^2^ Ischaemic stroke accounts for over 80% of all strokes. Transient or permanent ischaemia and hypoxia to specific regions of the brain bring about neuronal apoptosis and death, leading to localized brain damage and functional deficits.^3^, ^4^, ^5^ Neural stem cells (NSCs) are multipotent cells that have the capacity for self‐renewal, migration to specific sites and differentiation into three main central nervous system (CNS) lineages—neurons, astrocytes and oligodendrocytes. Ischaemic stroke has been reported to trigger NSCs to proliferate and migrate towards the brain infarct, where they subsequently differentiate into neurons.^6^ This process of endogenous neurogenesis is believed to contribute to post‐stroke functional recovery. Unfortunately, the endogenous regenerative response is limited and does not lead to full recovery after stroke.^7^, ^8^

Mesenchymal stem cells (MSCs) have emerged as powerful strategies to improve stroke recovery.^9^ Studies have reported that MSCs harvested from bone marrow (BMSCs) and adipose tissue (Ad‐MSCs) could enhance functional recovery and neurogenesis in stroke model.^10^, ^11^ Increasing evidence indicates that stem cells exert therapeutic effects primarily through paracrine actions.^12^ Exosomes are critical mediators of cell paracrine action. M. Chopp et al have previously demonstrated that MSC‐generated exosomes could enhance the stroke recovery process by promoting neurogenesis.^13^, ^14^, ^15^ However, the most commonly investigated MSCs, such as BMSCs and Ad‐MSCs, have a limited source and require invasive procedures to isolate, severely hampering their clinical applications. Our group isolated a stem cell population from voided urine, which can be conveniently obtained through a simple, non‐invasive and low‐cost approach.^16^, ^17^ Aside from their capacity for osteogenic, chondrogenic and adipogenic differentiation potential, we further found that urine‐derived stem cells (USCs) could also differentiate into neural lineage cells. After transplantation into rat brains, USCs could survive, migrate and differentiate into neuronal lineages at the lesion site.^18^ These characteristics make USCs a promising alternative to current MSCs in ischaemic stroke therapy. Nevertheless, few studies have directly utilized exosomes derived from human urine‐derived stem cells (USC‐Exos) for ischaemic stroke therapy. In the present study, we investigated whether USC‐Exos could promote neurogenesis and functional recovery in ischaemic stroke.

Endogenous neurogenesis involves a complex process beginning with the proliferation of NSCs, followed by the differentiation of NSCs into new neurons.^19^, ^20^ Epigenetic modification has emerged as a critical mechanism regulating gene expression in a temporal and spatial manner during proliferation and differentiation of NSCs.^21^, ^22^ Lysine acetylation of histones, which is one of the best characterized epigenetic modifications, is determined by the interplay between 2 classes of antagonistic enzymes, histone acetyltransferases (HATs) and histone deacetylases (HDACs).^23^ Recent observations point to a critical role for HDACs in the modulation of NSCs' self‐renewal and differentiation by controlling the activity of a network of downstream target genes.^24^, ^25^ Furthermore, pharmacological manipulation of HDAC activities using HDAC inhibitors (HDACis) such as valproic acid (VPA),^24^, ^26^ trichostatin A (TSA),^27^ suberoylanilide hydroxamic acid (SAHA)^28^ and sodium butyrate^26^, ^29^ promotes differentiation of NSCs into neural cells. HDAC6 is a member of HDAC family. The domain structure of HDAC6 is distinct from that of all other HDACs, as it harbours 2 deacetylase domains and a C‐terminal zinc finger domain.^30^ Previous research has demonstrated that experimental ischaemic stroke causes an induction of HDAC6 both in vivo and in vitro.^31^ Inhibition of HDAC6 significantly promoted neurogenesis and alleviated functional deficits following ischaemic injury.^32^, ^33^ Knock‐down of HDAC6 or suppression of HDAC6 level in neurons decreased neurite outgrowth and slowed axonal growth.^34^, ^35^ More importantly, HDAC6 inhibition could promote differentiation of NSCs.^36^ Given these observations, HDAC6 represents an interesting regulator in the process of neurogenesis after stroke.

In this study, we investigated the effects of USC‐Exos on neurogenesis in ischaemic stroke models, as well as the relationship between HDAC6 expression and USC‐Exos–induced neurogenesis. Our in vivo study indicated that USC‐Exos could promote endogenous neurogenesis and enhance the repair of neurological functions in post‐ischaemic stroke rats. We further found that USC‐Exos increased both proliferation and neuronal differentiation of oxygen‐glucose deprivation/reoxygenation (OGD/R)‐stimulated NSCs. A further mechanism study revealed that the pro‐neurogenesis effects of USC‐Exos may be associated with the repression of HDAC6 expression via translocation of exosomal microRNA‐26a (miR‐26a).

## 2 MATERIALS AND METHODS

### 2.1 Isolation and identification of USCs

Urine‐derived stem cells were isolated from the urine samples of healthy adult donors ranging in age from 24 to 28 years, according to the methods described in our previous studies.^16^, ^37^ Written informed consent was obtained from all of the volunteers. The study was conducted in accordance with the Declaration of Helsinki, and the protocol was approved by the Ethics Committee of the Shanghai Sixth People's Hospital affiliated with Shanghai Jiao Tong University. The expressions of USC surface marker proteins (Becton Dickinson), such as CD29‐PE, CD90‐FITC, CD73‐PE, CD44‐FITC, CD34‐APC and HLA‐DR‐PE, were measured using flow cytometry.

### 2.2 Isolation and identification of USC‐Exos

Exosomes were isolated from the USC supernatant by differential centrifugation/ultracentrifugation protocols. Briefly, the obtained medium was centrifuged at 300 *g* for 15 minutes and 2000 *g* for 30 minutes to remove dead cells and cellular debris. After centrifugation at 10 000 *g* for 1 hour, the supernatant was further ultracentrifuged at 100 000 *g* for 2 hours. After the removal of supernatant, the pellet was resuspended in phosphate buffer saline (PBS), followed by another ultracentrifugation at 100 000 *g* for 2 hours. Finally, pelleted exosomes were resuspended in PBS. Exosome morphologies were detected using transmission electron microscopy (TEM). Briefly, fresh exosome samples were loaded onto a continuous carbon grid, and then fixed in 3% (w/v) glutaraldehyde and stained with 2% uranyl acetate. The samples were then examined with a Hitachi H‐7650 transmission electron microscope (Hitachi). The size and concentration of the exosomes were assessed using flow nanoanalyser instruments according to the manufacturer's instructions.^38^, ^39^ Exosomal markers CD9 (1:1000; Abcam), TSG101 (1:1000; Abcam), Alix (1:1000; Abcam) and negative marker Golgi membrane bound protein (GM130；1:500; Abcam) were detected using Western blot analysis.

### 2.3 NSC culture

Subventricular zone NSCs were dissociated from Sprague‐Dawley (SD) rats and cultured as previously described.^40^ The proliferation medium of the NSCs was composed of Dulbecco's modified Eagle medium (DMEM)/F12 media (Gibco) supplemented with 2% (v/v) B27 (Gibco), 20 ng/mL epidermal growth factor (EGF; Perprotech), 20 ng/mL basic fibroblast growth factor (bFGF; Perprotech), 1% penicillin‐streptomycin (Gibco) and 2 μg/mL heparin (Sigma). Passages 2‐4 were used for the following experiments.

### 2.4 Application of oxygen‐glucose deprivation/reoxygenation (OGD/R)

To mimic ischaemic‐like conditions in vitro, the culture medium of the NSCs was replaced with glucose‐free DMEM containing the same supplements as those in the proliferation medium. The NSCs were then transferred to anaerobic conditions (5% CO_2_ and 95% N_2_) for 8 hours. OGD was then ended by changing the medium to glucose and returning the NSCs to normoxia cultured with either the absence (vehicle) or presence of USC‐Exos (1 × 10^9^ particles/mL) for 24 hours. Control NSCs were cultured under normal conditions without any treatment. Cells were then harvested for further analysis.

### 2.5 Assessment of NSC proliferation and neuron differentiation after OGD/R

The 5‐ethynyl‐2′‐deoxyuridine (EdU, 10 µmol/L, Invitrogen) was added to the culture medium following OGD/R. After an additional 24 hours, the cells were stained with Nestin antibody (1:100; Abcam) and EdU click reaction (Invitrogen). Immunoreactive cells were visualized using fluorescence microscopy (Leica). Differentiation of NSCs was induced by withdrawal of EGF and bFGF following OGD/R. At day 7, the differentiation was evaluated with Tuj1 (1:100; Abcam) staining. Each experiment was repeated at least three times.

### 2.6 Cell transfection

For HDAC6 overexpression, NSCs were transfected with HDAC6 plasmid (1 μg) using Lipofectamine 2000 (Invitrogen) in Opti‐MEM (Invitrogen) according to the manufacturer's instructions. HDAC6 overexpression plasmid and control vector plasmid were purchased from RiboBio.

For miR‐26a knock‐down, USCs were transfected with inhibitor control (IC) or miR‐26a inhibitor (100 nmol/L; RiboBio) using Lipofectamine RNAiMAX reagent (Invitrogen) according to the manufacturer's protocol. The transfected cells were cultured for 48 hours prior to use in subsequent experiments.

### 2.7 Cerebral ischaemia model and USC‐Exos injection

All of the animal study protocols were approved by the Animal Research Committee of the Shanghai Sixth People's Hospital (SYXK [Shanghai, China] 2011‐0128, 1 January 2011). Transient focal cerebral ischaemia was induced via middle cerebral artery occlusion (MCAO). SD rats (6‐8 weeks old, male, 250‐300 g) were subjected to 2 hours of right MCAO using an intraluminal suture vascular occlusion. Rats showing neither hemiplegia nor neurological deficits post‐MCAO were excluded for data analysis consistency. Animals were then randomized into four groups (n = 10/group): the sham + vehicle group, the sham + USC‐Exos group, the MCAO + vehicle group and the MCAO + USC‐Exos group. Rats in the sham group underwent the same procedure without vascular occlusion. Approximately 1 × 10^11^ total particles of USC‐Exos in 500 μL phosphate‐buffered saline (PBS) or vehicle (PBS alone, 500 μL) were administered via a single intravenous injection 4 hours post‐MCAO.

### 2.8 Uptake of USC‐exos in vitro

To determine whether USC‐Exos administered intravenously could migrate into the brain, USC‐Exos were stained with DiR fluorescent dye (Thermo Fisher), per the manufacturer's instructions. Four hours post‐MCAO, the rats were administered a single dose of DiR‐labelled exosomes (1 × 10^11^ particles in 500 μL PBS). Six hours later, the DiR‐related fluorescence signals were detected using the IVIS Spectrum imaging system (PerkinElmer).

In vitro, USC‐Exos were labelled with Dil fluorochrome (Thermo Fisher) according to the manufacturer's instructions and incubated with NSCs for 4 hours. The signals were analysed using a fluorescence microscope (Leica).

### 2.9 Infarct volume assessment

Brain infarct volume was measured by magnetic resonance imaging (MRI) or cresyl violet staining. For brain MRI study, a 3‐Tesla magnetic resonance imaging (MRI) scanner (Siemens) was used to assess the infarct volume at 2 and 28 days post‐MCAO. The hyperintense areas in the T2‐weighted images over the central 8 images (1.5‐mm thick) were used to calculate the infarct volume. The infarct volume of the brain was quantified using ImageJ software (NIH). For cresyl violet staining, infarct volume of was calculated by adding up the infarct areas (contralateral area minus ipsilateral side non‐infarct area) of six consecutive slices using ImageJ software (NIH).

### 2.10 Behaviour test

The modified neurological severity score (mNSS) and the foot‐fault test were performed pre‐MCAO and 1, 3, 7, 14, 21 and 28 days post‐MCAO, as described previously.^41^

### 2.11 Brain tissue preparation and immunofluorescence

EdU was injected (50 mg/kg) 24 hours before MCAO. On the day 14 after MCAO, the rats were anaesthetized and fixed by transcardiac perfusion with 4% paraformaldehyde. Brains were removed, fixed in 4% formaldehyde overnight at 4°C and immersed in 30% sucrose for 72 hours for cryoprotection. After being embedded and frozen in an optimal cutting‐temperature compound, the brains were sliced into 20‐μm‐thick coronal sections. The brain sections were then stained with specific markers to Nestin (1:100, Abcam), Sox2 (1:100, Cell Signaling Technology), doublecortin (DCX, 1:100) and NeuN (1:200, Cell Signaling Technology) with EdU click reaction (Thermo Fisher). Images were obtained using a Leica microscope (Leica).

### 2.12 Western blot analysis

Western blot analysis was performed according to routine protocols. Primary antibodies used were β‐actin (1:1000; Abcam) and HDAC6 (1:1000; Cell Signaling Technology). The intensity of each band was analysed using ImageJ software.

### 2.13 Quantitative reverse‐transcription PCR (qRT‐PCR)

The miR‐26a expression was detected with a mirVana qRT‐PCR miRNA Detection kit (Thermo Fisher Scientific) that included the miR‐26a‐specific RT primer and quantitative primers, reverse transcriptase and reaction mix. The relative expression of miRNAs was evaluated using the 2^−ΔΔCt^ method and normalized to U6. Reactions were performed in triplicate, and independent experiments were repeated three times.

### 2.14 Statistical analysis

Each experiment was repeated at least three times, and the data were presented as mean ± SEM. Statistical analysis was performed using GraphPad Prism 7.0 software. Student's *t* test and the one‐way analysis of variance (ANOVA) with Bonferroni post hoc Tukey test were respectively applied for comparisons between two groups and three groups or more. Statistical significance was considered to be *P* < .05.

## 3 RESULTS

### 3.1 Characterization of USCs and USC‐Exos

**Figure 1:**
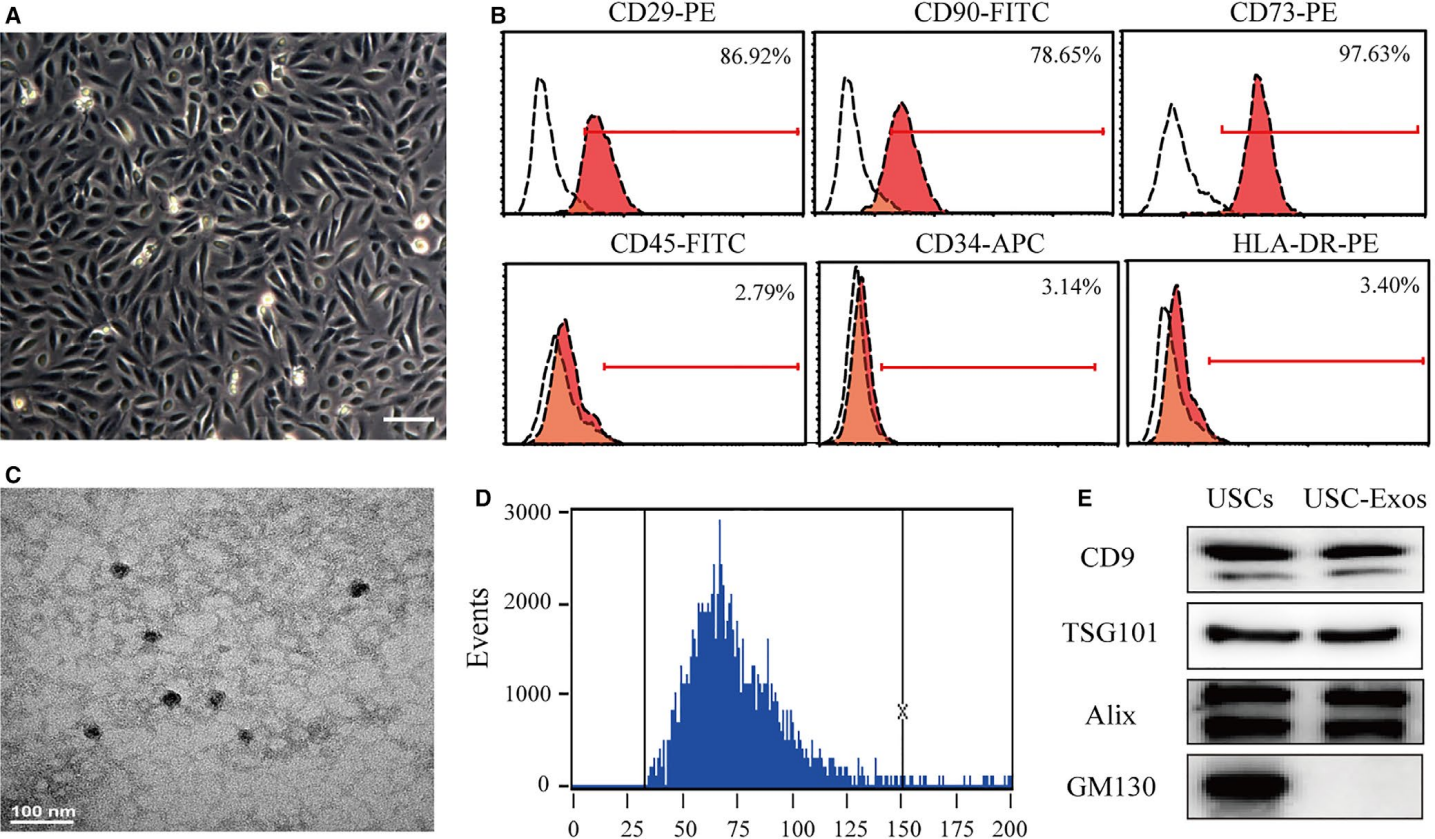
Characterization of human urine‐derived stem cells (USCs) and USC‐derived exosomes (USC‐Exos). A, The morphology of USCs was observed under a microscope. Scale bar = 50 μm. B, Flow cytometric analysis of the expression of cell surface markers on USCs. USCs were positive for CD29, CD90 and CD73, and negative for CD45, CD34 and HLA‐DR. C, Representative transmission electron microscopic (TEM) image of USC‐Exos. Scale bar = 100 nm. D, The size distribution of the USC‐Exos was examined using flow nanoanalyser analysis. The mean diameter of USC‐Exos was 74.2 ± 16 nm. E, The positive markers for exosomes—CD63, TSG101 and Alix—were detected in USC‐Exos using Western blot analysis, whereas the negative marker GM130 was not detected

Urine‐derived stem cells in primary culture presented a fibroblast‐like morphology (Figure 1A). Cell surface markers were assessed using cytometric analysis. As shown in Figure 1B, USCs were positive for CD29, CD90 and CD73, and negative for CD45, CD34 and HLA‐DR. Exosomes were isolated from USC culture supernatant and characterized using TEM, a flow nanoanalyser (NanoFCM) and Western blot analysis. The results of TEM showed that USC‐Exos were spherical vesicles with diameters ranging from approximately 30‐100 nm (Figure 1C). Flow nanoanalyser analysis revealed that the average diameter was 74.2 ± 16 nm (Figure 1D), and the concentration of the USC‐Exos was approximately 2 × 10^11^ particles/mL. Western blot analysis revealed that USC‐Exos expressed exosomal markers, such as CD9, TSG101 and Alix, whereas the negative marker GM130 was not detected (Figure 1E). These data suggested that we had successfully isolated USC‐Exos.

### 3.2 USC‐Exos reduce infarct volume and promote functional recovery in rats after ischaemic stroke

**Figure 2:**
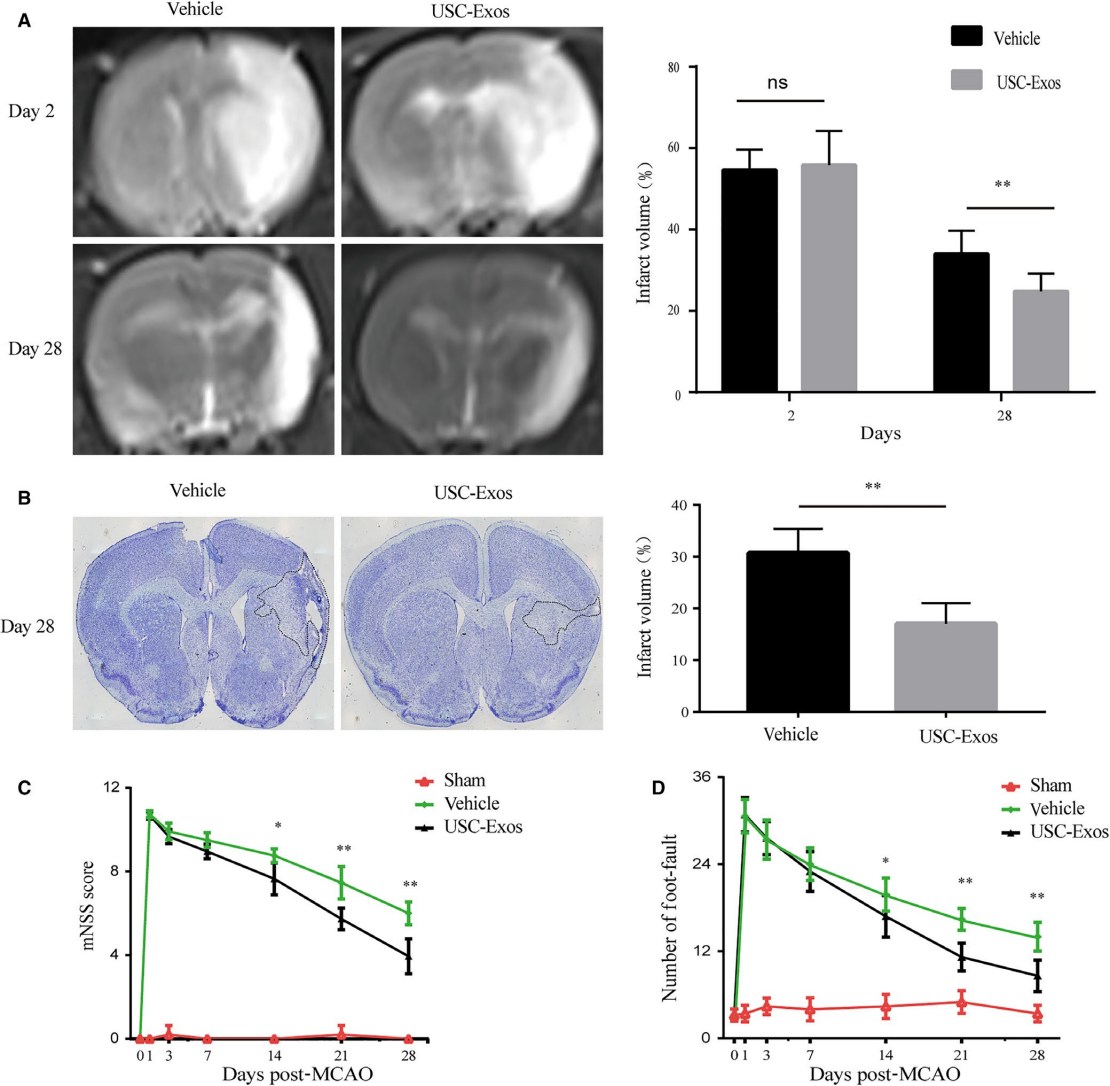
USC‐Exos reduce infarct volume and promote functional recovery in rats after ischaemic stroke. A, Brain infarct volumes in each group were measured using magnetic resonance imaging (MRI) at day 2 and day 28 post‐stroke. B, Infarct volume was measured by *cresyl violet staining* 28 d after MCAO. The modified neurological severity score (mNSS) and the foot‐fault test were performed pre‐MCAO and 1, 3, 7, 14, 21 and 28 d post‐MCAO. Statistical analysis of (C) mNSS score and (D) foot‐fault test in each group. n = 10/group. *P < .05, **P < .01; *ns* = not significant

To determine whether the intravenous infusion of USC‐Exos could migrate into the brain, USC‐Exos were labelled with DiR fluorescent dye and administered to rats at 4 hours after MCAO. DiR‐labelled USC‐Exos–treated rats exhibited visibly high levels of fluorescence intensity in the brain compared to vehicle‐treated rats, confirming that USC‐Exos could cross the blood‐brain barrier and migrate into the brain (Figure S1A). We then further investigated the therapeutic effects of USC‐Exos in rats after ischaemic stroke. Brain infarct size was monitored using MRI at day 2 and day 28 post‐MCAO (Figure 2A). There was no difference in the original infarct volume measured in the two groups at day 2 post‐stroke. However, the infarct volume had significantly decreased in the USC‐Exos group compared with the vehicle group at day 28 post‐stroke. We also performed cresyl violet staining 28 days after MCAO, which in consistent with MRI result, showed that USC‐Exos injection significantly attenuated brain infarct 28 days after MCAO (Figure 2B). Measurements from behavioural tests were then used to examine whether USC‐Exos treatment led to long‐term improvement in behavioural function. As expected, USC‐Exos treatment significantly increased functional improvement in both the mNSS (Figure 2C) and the foot‐fault test (Figure 2D) starting 14 days post‐stroke compared with the vehicle group. These results indicate that USC‐Exos ameliorate functional outcome in rats after cerebral ischaemic injury.

### 3.3 USC‐Exos promote neurogenesis in rats after ischaemic stroke

**Figure 3:**
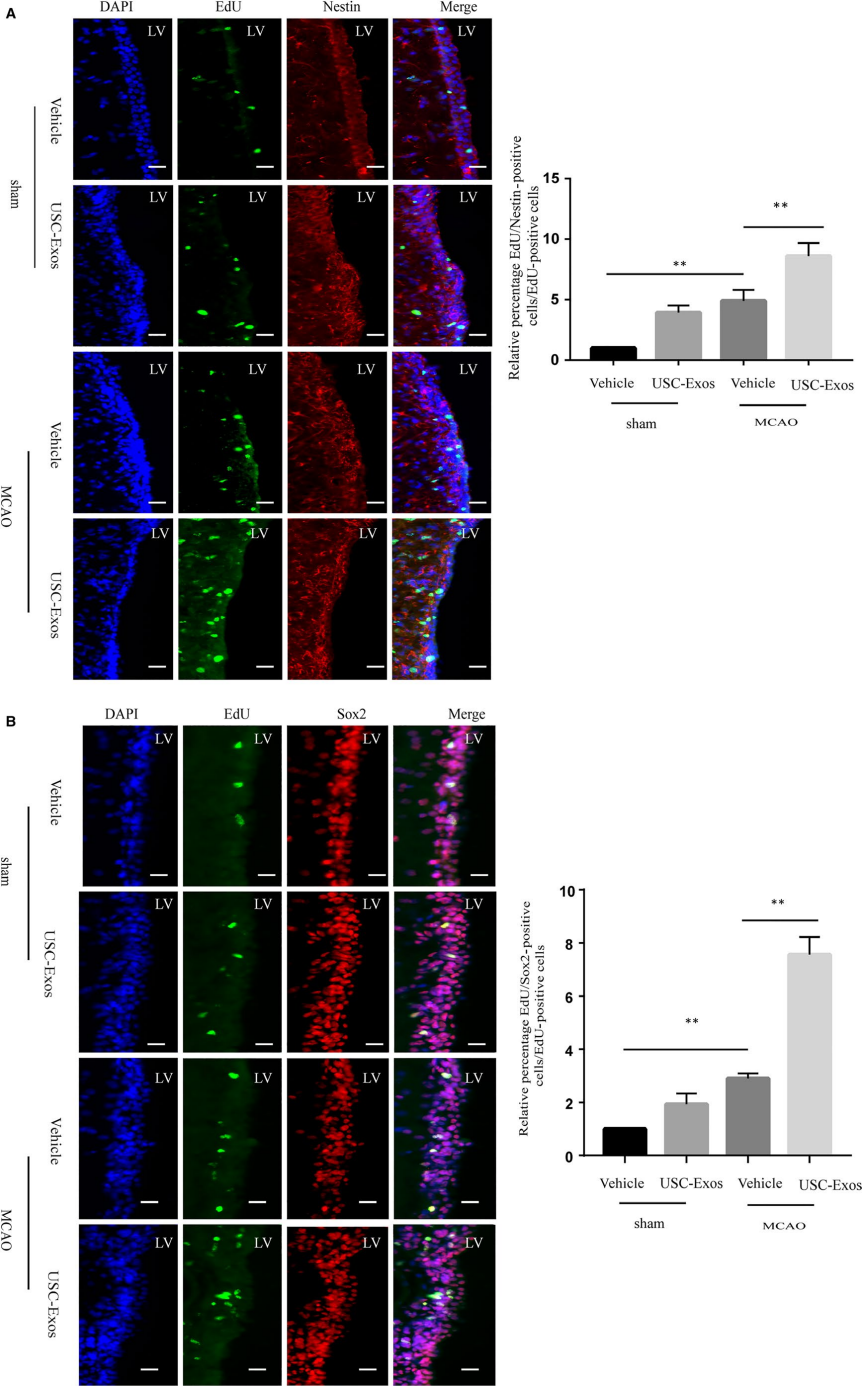
USC‐Exos promote SVZ NSC proliferation in rats after ischaemic stroke. A, Representative immunofluorescent staining images and quantitative analysis of EdU (green) and Nestin (red) double‐positive cells in the subventricular zone (SVZ) of rat brains at 14 d post‐ischaemic stroke. B, Representative immunofluorescent staining images and quantitative analysis of EdU (green) and Sox2 (red) double‐positive cells in the subventricular zone (SVZ) of rat brains at 14 d post‐ischaemic stroke. Scale bar = 100 μm. n = 6/group. *P < .05, **P < .01

**Figure 4:**
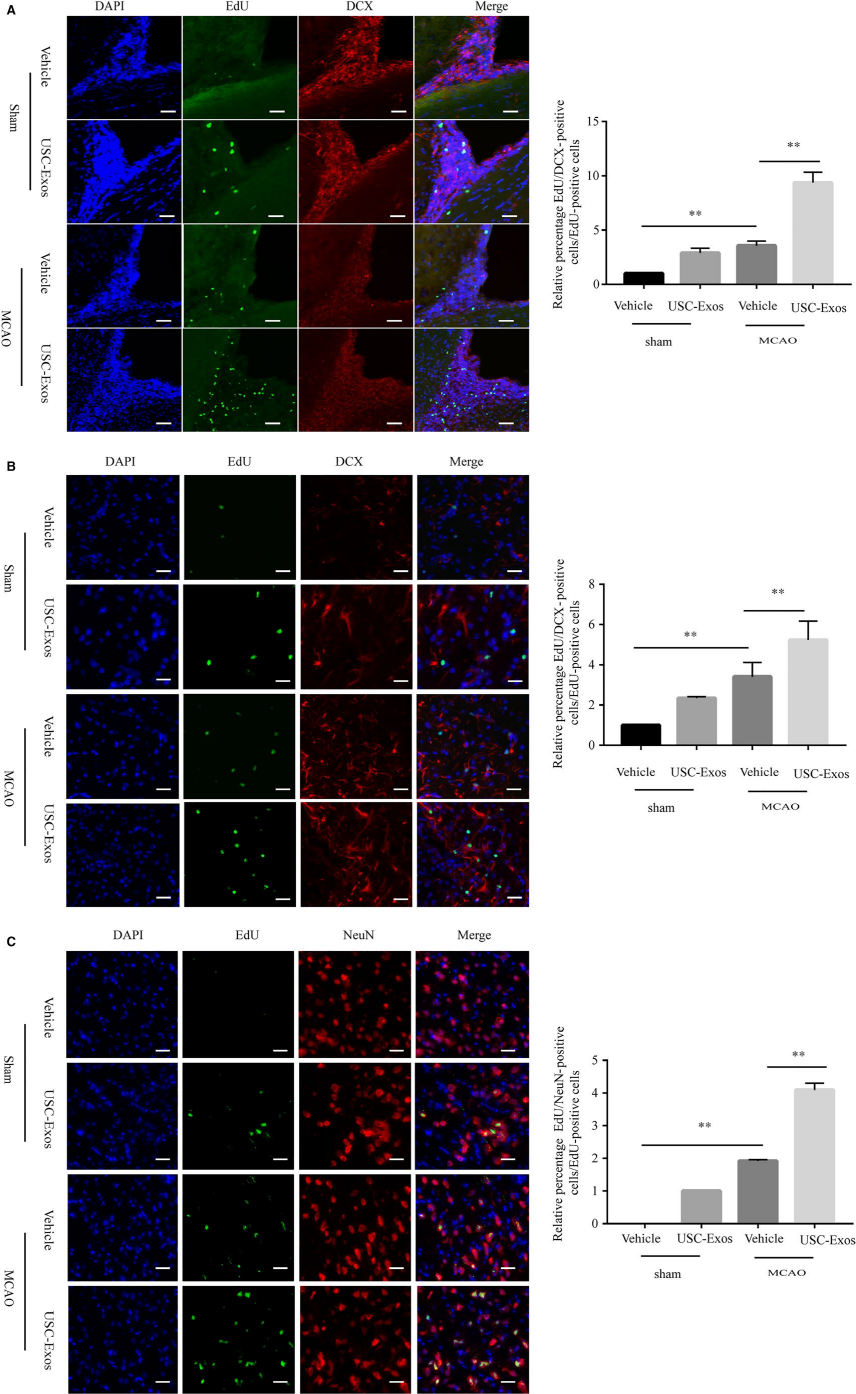
USC‐Exos promote SVZ NSC differentiation in rats after ischaemic stroke. A, Representative immunofluorescent staining images and quantitative analysis of EdU (green) and DCX (red) double‐positive cells in the subventricular zone (SVZ) of rat brains at 14 d post‐ischaemic stroke. B, Representative immunofluorescent staining images and quantitative analysis of EdU (green) and DCX (red) double‐positive cells in the ischaemic brain boundary zone of rat brains at 14 d post‐ischaemic stroke. C, Representative immunofluorescent staining images and quantitative analysis of EdU (green) and NeuN (red) double‐positive cells in the ischaemic brain boundary zone of rat brains at 14 d post‐ischaemic stroke. Scale bar = 100 μm. n = 6/group. * P < .05, ** P < .01; *ns* = not significant

We then examined whether USC‐Exos could enhance neurogenesis after ischaemic stroke in rats. The subventricular zone (SVZ) lining the lateral ventricle (LV) is a major area in which adult NSCs reside, thus making it a potential therapeutic target that may allow neurogenesis. To evaluate the effects of USC‐Exos on the proliferation of SVZ NSCs, we quantified EdU^+^/Nestin^+^ or EdU^+^/Sox2^+^ cells at 14 days post‐MCAO. As shown in Figure 3A,B, USC‐Exos stimulated SVZ EdU^+^/Nestin^+^ or EdU^+^/Sox2^+^ cells in sham rats. Furthermore, the vehicle group had significantly higher rates of EdU^+^/Nestin^+^ or EdU^+^/Sox2^+^ cells in the SVZ than the sham control group, indicating the spontaneous proliferation of NSCs in the ischaemic brain. Notably, the USC‐Exos treatment group had remarkably high levels of EdU^+^/Nestin^+^ or EdU^+^/Sox2^+^ staining cells compared to the vehicle group, indicating that USC‐Exos promote the proliferation of NSCs in the SVZ of post‐MCAO rats. We then investigated whether USC‐Exos could promote neuronal differentiation of SVZ NSCs at 14 days post‐MCAO. As shown in Figure 4A, USC‐Exos stimulated SVZ EdU^+^/DCX^+^ cells both in sham and post‐MCAO rats. To evaluate whether SVZ EdU^+^/DCX^+^‐positive cells could migrate into the damaged brain and then differentiate into mature neurons, we detected EdU^+^/DCX^+^ or EdU^+^/NeuN^+^ cells in the infarct boundary zone at 14 days post‐MCAO. USC‐Exos markedly increased EdU^+^/DCX^+^ or EdU^+^/NeuN^+^ cells in the infarct boundary zone in post‐MCAO rats (Figure 4B). These results suggest that USC‐Exos promote neurogenesis in rats after stroke, which contributes to improving functional outcome.

### 3.4 USC‐Exos promote proliferation and neuronal differentiation of NSCs subjected to OGD/R

The characteristics of NSCs were identified prior to the following experiments. NSCs cultured in vitro formed spherical free‐floating neurospheres (Figure S2A). Immunocytochemistry staining revealed that most of the cells in the neurospheres were Nestin/Sox2‐positive cells (Figure S2B). These characteristics indicate that the cultured neurospheres have phenotypes of NSCs. We then determined whether USC‐Exos could be internalized by NSCs. USC‐Exos were labelled with Dil fluorescent dye and added to NSC culture media. After 4 hours of incubation, Dil‐labelled USC‐Exos were efficiently internalized by NSCs (Figure S2C).

**Figure 5:**
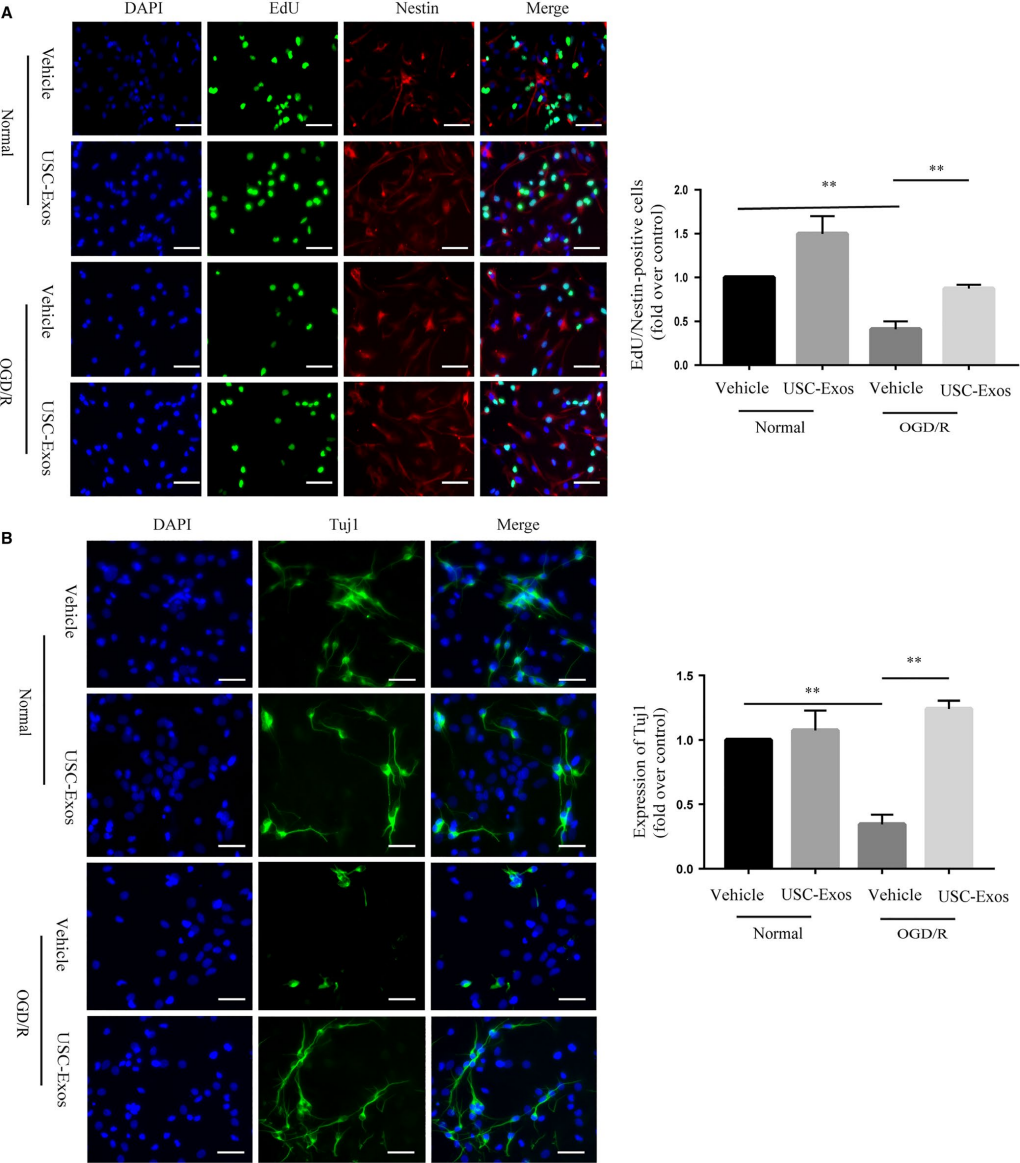
USC‐Exos promote proliferation and neuronal differentiation of NSCs subjected to OGD/R. A, Representative immunofluorescent staining images and quantitative analysis of EdU (green) and Nestin (red) double‐positive cells in each group. B, Representative immunofluorescent staining images and quantitative analysis of Tuj1 (green)‐positive cells in each group. Scale bar = 100 μm. *P < .05,**P < .01

The effects of USC‐Exos on the proliferation and neuronal differentiation of NSCs after OGD/R were investigated using immunofluorescence. As shown in Figure 5A, USC‐Exos stimulated EdU^+^/Nestin^+^ cells in normal NSCs. Next, we subjected the NSCs to an ischaemia‐like insult, OGD/R, which induced a significant decrease in the number of EdU^+^/Nestin^+^ cells compared with normal NSCs. Treatment with USC‐Exos obviously increased the numbers of EdU^+^/Nestin^+^ cells as compared to the OGD/R group. After being subjected to OGD/R, NSCs were cultured in a differentiation medium. After 7 days of differentiation, cells were stained with Tuj‐1. As shown in Figure 5B, USC‐Exos increased Tuj‐1^+^ cells in normal NSCs. Compared to the control group, the percentage of Tuj‐1^+^ cells decreased after OGD/R treatment. The percentage of Tuj‐1^+^ cells significantly increased after treatment with USC‐Exos compared with the vehicle group. These results indicate that USC‐Exos promote the proliferation and neuronal differentiation of NSCs post‐OGD/R.

### 3.5 USC‐Exos increase proliferation and neuronal differentiation of OGD/R‐stimulated NSCs by inhibiting HDAC6 expression

**Figure 6:**
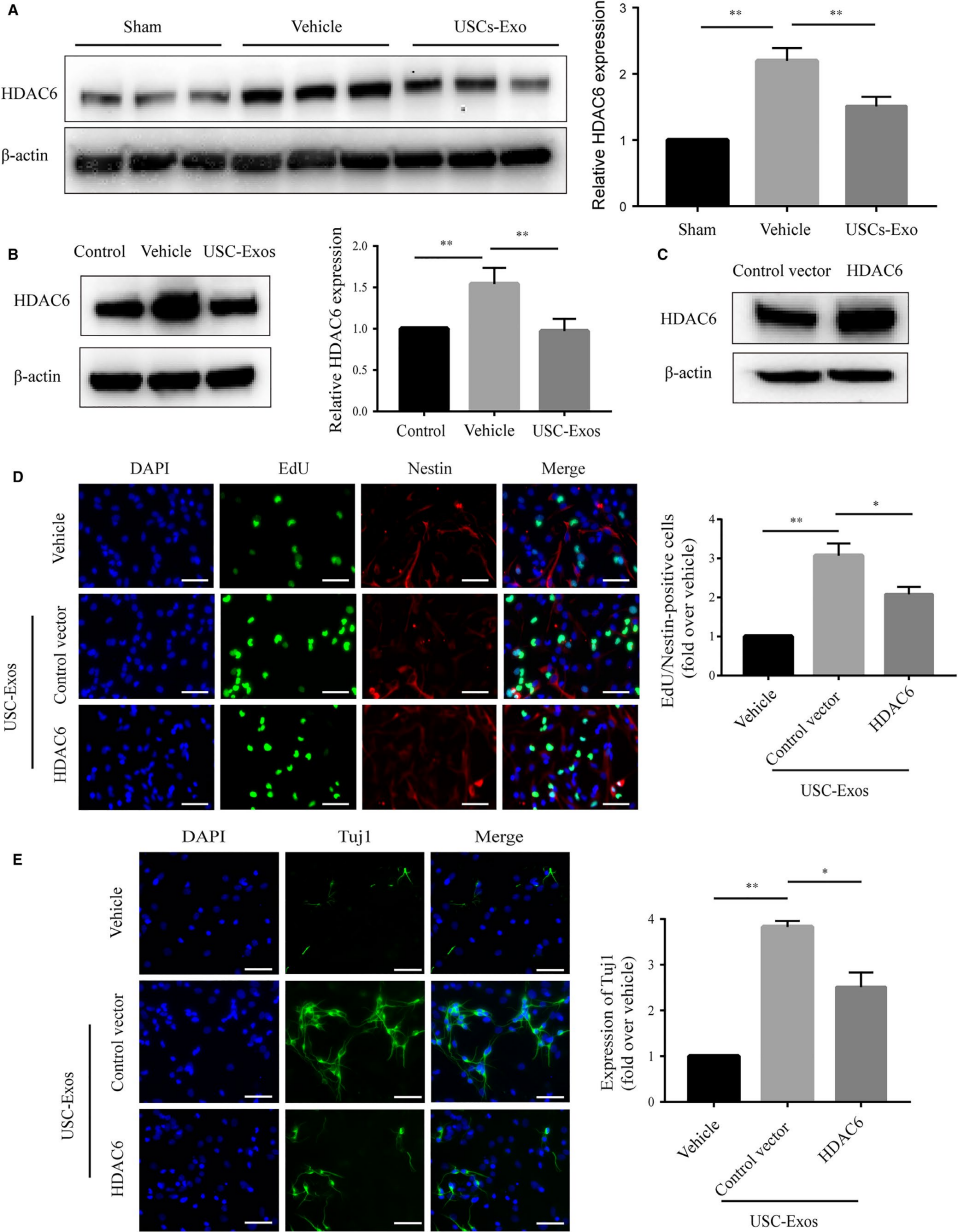
USC‐Exos increase proliferation and neuronal differentiation of OGD/R‐stimulated NSCs by inhibiting HDAC6 expression. A, Protein levels of HDAC6 were measured using Western blot analysis and then quantified with a grey value assay in the peri‐infarct brains at 14 d after stroke in each group. n = 3/group. B, Protein levels of HDAC6 were measured using Western blot analysis and then quantified with a grey value assay of NSCs subjected to OGD/R in each group. NSCs subjected to OGD/R were transfected with control vector plasmid or HDAC6 overexpression plasmid, and then, the downstream experiments were performed. C, The efficiency of HDAC6 overexpression plasmid was tested by measuring HDAC6 protein expression using Western blot analysis. D, Immunofluorescence and quantification of EdU (green) and Nestin (red) double‐positive cells in different treatment groups. E, Immunofluorescence and quantification of Tuj1 (green)‐positive cells in different treatment groups. Scale bar = 100 μm. *P < .05, **P < .01

We then investigated the relationship between HDAC6 expression and the effects of USC‐Exos–induced pro‐neurogenesis activity in ischaemic stroke. The protein expression of HDAC6 was evaluated in ischaemic rat brains 14 days post‐MCAO. As shown in Figure 5A, the expression of HDAC6 was significantly higher in the vehicle group than in the sham group. The expression level of HDAC6 in the USCs‐Exo group was significantly lower than that of the vehicle group. We then confirmed the expression of HDAC6 in NSCs subjected to OGD/R (Figure 6B). As expected, the expression of HDAC6 increased after OGD/R. Incubating with USCs‐Exo significantly down‐regulated the expression of HDAC6 in NSCs subjected to OGD/R. In order to further determine whether HDAC6 was involved in the process of USC‐Exos‐mediated pro‐neurogenesis, an overexpression HDAC6 gene plasmid was transfected into NSCs subjected to OGD/R, and then, NSCs were stimulated with or without USC‐Exos for 24 hours. Western blot analysis confirmed the up‐regulation efficiency of HDAC6 in NSCs transfected with the HDAC6 overexpression plasmid in the absence of USC‐Exos treatment (Figure 6C). NSCs overexpressing HDAC6 were then subjected to functional assays to measure cell proliferation and neuronal differentiation. Strikingly, HDAC6 overexpression abated the promotion of proliferation and neuronal differentiation in response to treatment with USC‐Exos in NSCs subjected to OGD/R (Figure 6D,E). These data indicate that USC‐Exos–induced proliferation and neuronal differentiation in NSCs after OGD/R may be partially attributed to HDAC6 inhibition.

### 3.6 miR‐26a shuttled by USC‐Exo increase proliferation and neuronal differentiation of OGD/R‐stimulated NSCs by targeting HDAC6

**Figure 7:**
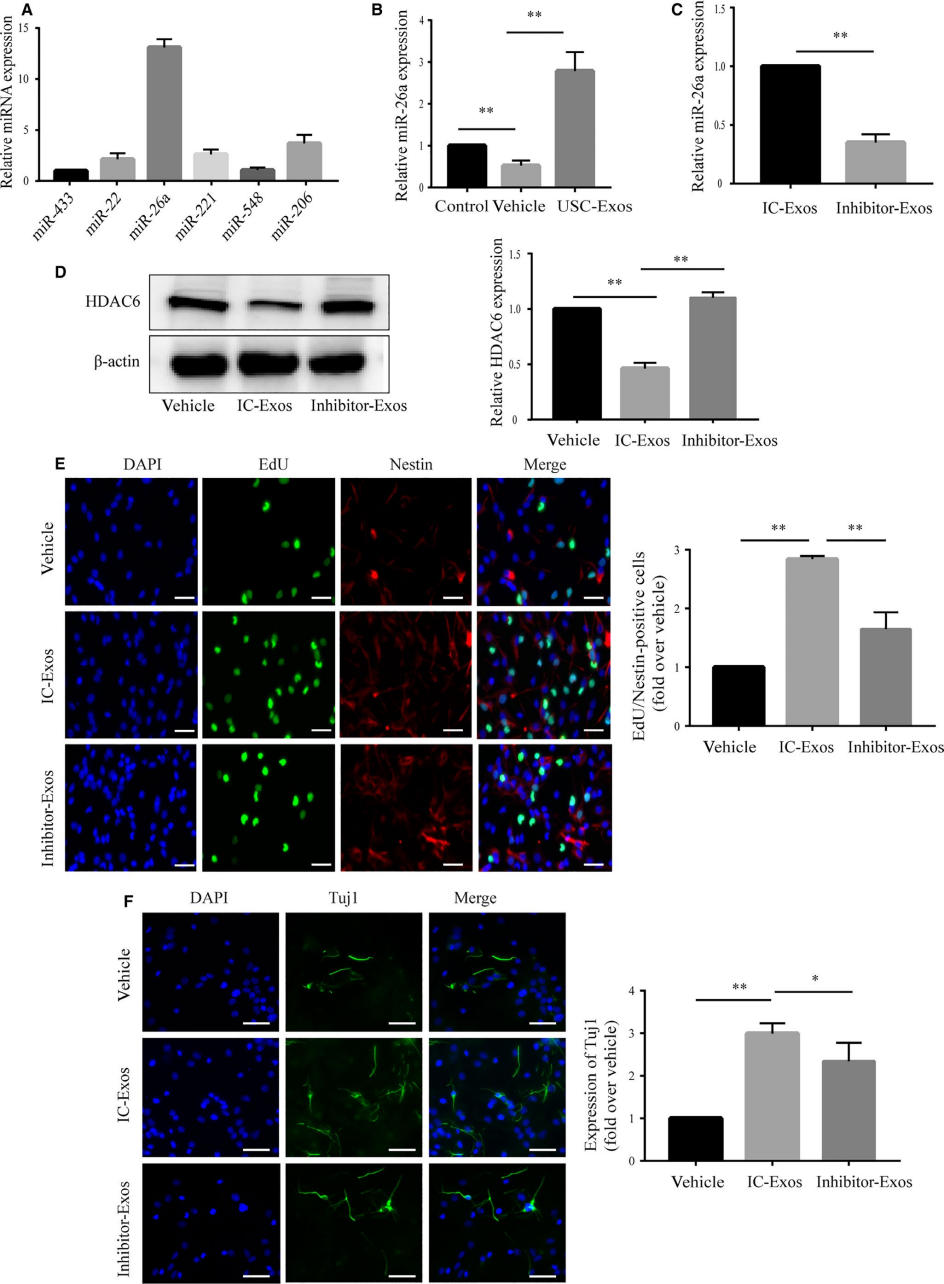
miR‐26a shuttled by USC‐Exo increase proliferation and neuronal differentiation of OGD/R‐stimulated NSCs by targeting HDAC6. A, RT‐qPCR analysis showed that miRNAs reported to be HDAC6 regulators, including miR‐433, miR‐221, miR‐26a, miR‐22, miR‐548 and miR‐206, are contained in USC‐Exos. B, Exosomes were isolated from USCs transfected with 100 nmol/L inhibitor (inhibitor‐Exos) or inhibitor control (IC‐Exos) as indicated. MiR‐26a expression was analysed in IC‐Exos or inhibitor‐Exos by RT‐qPCR. C, Expression levels of miR‐26a in different treatment groups. D, Expression of HDAC6 in different treatment groups. E, Immunofluorescence and quantification of EdU (green) and Nestin (red) double‐positive cells in different treatment groups. F, Immunofluorescence and quantification of Tuj1 (green)‐positive cells in different treatment groups. Scale bar = 100 μm. *P* < .05, **P < .01

Exosomes mediate cell‐to‐cell communication by transferring their contents, especially miRNAs. We therefore have been suggested that USC‐Exos may affect the expression of HDAC6 by delivering exosomal miRNAs. We detected several miRNAs, including miR‐433,^42^ miR‐22,^43^, ^44^ miR‐26a,^45^ miR‐221,^46^ miR‐548^47^ and miR‐206,^48^ which had been reported to be HDAC6 regulators previously. We found that all of these miRNAs were contained in USC‐Exos. In particular, we discovered a higher expression level of miR‐26a in USC‐Exos (Figure 7A). Furthermore, the expression of miR‐26a in NSCs after incubation with USC‐Exos was determined by qRT‐PCR and markedly higher miR‐26a expression was detected in USC‐Exos–treated group compared with vehicle‐treated group, suggesting that miR‐26a may be transferred into NSCs through USC‐Exos (Figure 7B). To verify the role of miR‐26a in the USC‐Exos‐mediated promotion of NSC proliferation and neuronal differentiation, USCs were transfected with miR‐26a inhibitor or IC, and miR‐26a expression was analysed in exosomes isolated from these USC strains (inhibitor‐Exos or IC‐Exos). A significant decrease in miR‐26a expression was detected in inhibitor‐Exos compared with IC‐Exos (Figure 7C). Next, inhibitor‐Exos or IC‐Exos were incubated with NSCs to investigate the effects of exosomal‐transferred miR‐26a on HDAC6 expression in NSCs after OGD/R. As illustrated in Figure 7D, inhibitor‐Exos markedly reversed the USC‐Exos–induced reduction of HDAC6 in NSCs after OGD/R. Furthermore, the effects of exosomal‐transferred miR‐26a on OGD/R‐stimulated NSC proliferation and neuronal differentiation were also evaluated. NSC proliferation and neuronal differentiation were decreased following treatment with inhibitor‐Exos compared with IC‐Exos (Figure 7E,F). These findings indicate that the transfer of exosomal miR‐26a to NSCs through the targeting of HDAC6 may be one of the mechanisms by which USC‐Exos promote neurogenesis.

## 4 DISCUSSION

In this study, we found that USC‐Exos could significantly attenuated infarct volume and alleviate neurological deficits in post‐ischaemic stroke rats. Furthermore, USC‐Exos significantly increased the number of NSCs and newly differentiated neurons in post‐MCAO rats. Moreover, USC‐Exos promoted both proliferation and neuronal differentiation of NSCs subjected to OGD/R. Mechanistically, miR‐26a delivered by USC‐Exos may serve as a critical mediator in USC‐Exos–induced pro‐neurogenesis effects through targeting HDAC6. To our knowledge, this is the first report demonstrating that USC‐Exos enhance proliferation and neuronal differentiation of NSCs in ischaemic stroke, at least in part via the transfer of exosomal miR‐26a by HDAC6 inhibition.

Endogenous neurogenesis is known to contribute to repair and recovery in ischaemic stroke. However, this stroke‐induced neurogenesis needs to be further increased to attain more neural repair for functional recovery.^7^, ^20^ Exosome‐based cell‐free therapy, which overcomes the cell‐associated limitations in stem cell therapy, could be used as an alternative approach to stem cell infusion methods in the treatment of stroke.^49^ USC‐Exos are receiving much more attention for tissue repair. In the present study, we focused on the promotional effects of USC‐Exos on neurogenesis after stroke. NSC proliferation is the basic event for neurogenesis. In the current study, EdU labelling indicated that USC‐Exos significantly enhance NSC proliferation both in vitro and in vivo. The differentiation of NSCs into new neurons plays a key role in the long‐term functional recovery after ischaemic stroke. When studied in vivo, there was a significant increase in the number of EdU^+^/DCX^+^ or EdU^+^/NeuN^+^ cells after USC‐Exos treatment, demonstrating that USC‐Exos enhanced the formation and migration of new neurons. Our in vitro evidence indicated that USC‐Exos significantly enhance neuronal differentiation of NSCs subjected to OGD/R conditions mimicking the in vivo microenvironment after ischaemic stroke. These observations suggested that USC‐Exos increased NSC proliferation and neuronal differentiation, eventually contributing to the improved neurological outcome after ischaemic stroke.

Histone deacetylases‐mediated transcriptional repression is essential for NSC self‐renewal and differentiation by interacting with various cell‐intrinsic transcription factors and signalling pathways.^27^ HDAC inhibition regulates proliferation and neuronal differentiation of NSCs with up‐regulating expression of p21 and Pten, and neuronal‐specific genes, such as NeuroD, neurogenin 1 (Ngn1), Math1, cyclin D1 and B‐lymphocyte translocation gene 3.^24^, ^25^, ^28^ HDAC6 deacetylates various substrates including α‐tubulin and HSP90α, and is involved in many important biological processes, including cell proliferation and differentiation.^50^ Increasing evidence has demonstrated that HDAC6 represents a promising target in the development of therapeutic strategies targeting neurogenesis for neurological diseases such as stroke.^30^, ^51^ In this study, we explored the role of HDAC6 in USC‐Exos‐mediated neurogenesis. Our study found that the expression of HDAC6 significantly increased both in vitro and in vivo ischaemic stroke models. USC‐Exos could reduce the expression of HDAC6 induced by stroke models. Furthermore, overexpression of HDAC6 alleviated the pro‐neurogenesis effects induced by USC‐Exos in NSCs subjected to OGD/R. Therefore, HDAC6 inhibition may be the underlying mechanism by which USC‐Exos–induced neurogenesis in ischaemic stroke.

miRNAs are a group of 20‐24 nt small non‐coding RNAs which can regulate target gene expression by inducing mRNA degradation or translational inhibition. miRNAs have been confirmed to be selectively packaged into exosomes, and exosomes can transfer these miRNAs to target cells or tissues to regulate gene expression.^52^, ^53^ MiR‐26a was found to target HDAC6 in myogenic differentiation of embryonic stem cells.^45^ More importantly, studies have demonstrated that miRNA‐26a positively influenced stroke outcome.^54^, ^55^ Here, we detected high level of miR‐26a in USC‐Exos. After treated with USC‐Exos for 24 hours, we found that the expression of miR‐26a was significantly increased in NSCs subjected to OGD/R, indicating that miR‐26a can be transferred into NSCs through USC‐Exos. Furthermore, results of our study revealed that the effects of HDAC6 inhibition and pro‐neurogenesis induced by USC‐Exos were notably reversed by miR‐26a inhibitor‐Exos in vitro. These findings suggest that miR‐26a may be one of the critical mediators in USC‐Exos–mediated pro‐neurogenesis via targeting HDAC6. However, our present results could not exclude the possibility that other substances in USC‐Exos, such as other miRNAs, proteins or lipids, may also play roles in USCs‐Exo–induced neurogenesis by regulating HDAC6. Further investigation is also required to verify the role of miR‐26a/HDAC6 axis in USC‐Exos–mediated neurogenesis in vivo.

In summary, our research for the first time confirms that USC‐Exos exalt both proliferation and neuronal differentiation of cultured NSCs subjected to OGD/R, and promote neurogenesis in rats after stroke, potentially through miR‐26a/HDAC6 axis. Our finding of USC‐Exos promoting neurogenesis may open a new avenue for stroke treatment.

## CONFLICT OF INTEREST

The authors confirm that there are no conflicts of interest.

## AUTHORS' CONTRIBUTIONS

ZD and YW conceived the study, designed the experiments and provided their funds to the study. X. L, XN and QL participated in the experiments of USC identification and expansion. X. L, G. Z and QZ performed the in vivo experiments; X. L, G. Z and GH performed the in vitro experiments. X. L, G. Z, JZ and YY analysed the data. X. L, G. Z, ZD and YW wrote and revised the manuscript. All authors read and approved the final manuscript.

## Data Availability

The data used to support the findings of this study are included within the article.
