# Supplementary figures and images for: Exosomes from human urine‐derived stem cells enhanced neurogenesis via miR‐26a/HDAC6 axis after ischaemic stroke
Source: J Cell Mol Med. 2019 Oct 31;24(1):640–54. doi: 10.1111/jcmm.14774 (PMC6933407; doi:10.1111/jcmm.14774)

**FIGURE S1**


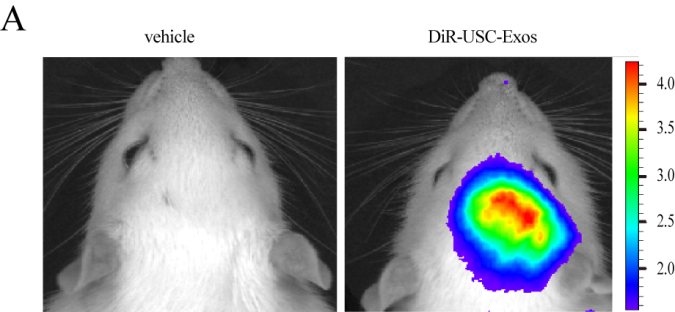


**FIGURE S2**

**
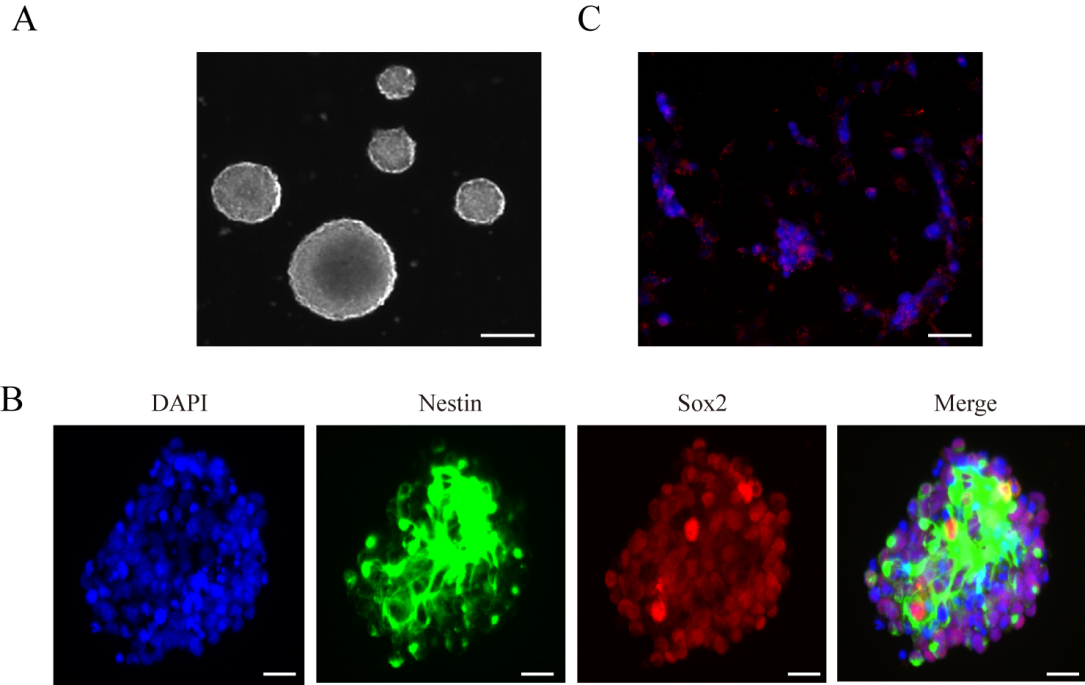
**

Supplement: Supplementary file 1 [file JCMM-24-640-s001.docx]
